# Sequential exposure to histological chorioamnionitis and postnatal hyperoxia alters gene expression in cord blood monocytes

**DOI:** 10.3389/fped.2026.1896464

**Published:** 2026-09-15

**Authors:** Rochelle Sequeira Gomes, Suhita Gayen Nee’ Betal, Sankar Addya, Saurabh Gayen, Nida Zubair, Joanna S. Y. Chan, Zubair H. Aghai

**Affiliations:** 1 Division of Neonatology, Nemours Children’s Health at Thomas Jefferson University, Philadelphia, PA, United States; 2 Cancer Genomics and Bioinformatics Laboratory, Sidney Kimmel Cancer Center, Thomas Jefferson University, Philadelphia, PA, United States; 3 Department of Chemistry and Physics, Lincoln University, PA, United States; 4 Department of Pathology, Thomas Jefferson University, Philadelphia, PA

**Keywords:** chorioamnionitis, hyperoxia, innate immunity, monocyte, neonatology

## Abstract

**Background:**

Sequential exposure to heterologous noxious stimuli such as histological chorioamnionitis (HCA) and hyperoxia may elicit variable immune responses, resulting in distinct disease manifestations in the neonate. However, the molecular mechanisms underlying innate immune dysregulation in these contexts remain poorly characterized.

**Methods:**

We conducted a pilot prospective observational study evaluating global gene expression in cord blood monocytes obtained from term infants born to mothers with and without HCA. Monocytes were exposed *in vitro* to hyperoxia (95% FiO_2_) or normoxia (21% FiO_2_) for 18–20 h. Transcriptome profiling was performed using Affymetrix Human Transcriptome Clariom-S chips. Differentially expressed genes were identified (fold change ≥1.5 and *p* value ≤ 0.05) and used for ingenuity pathway analysis to discover the altered canonical pathways, upstream regulators, networks, and disease associations involved.

**Results:**

We studied monocytes from 14 neonates (HCA *n* = 7, no HCA *n* = 7). Compared with no HCA–Normoxia, HCA alone exhibited 1,195 differentially expressed genes, with transcriptional signatures consistent with monocyte activation and differentiation (including *CLEC5A, FCGR3A, FCGR2B, CD101, PRAM1*, and *FN1* upregulation). Hyperoxia alone yielded 533 differentially expressed genes, while sequential exposure to HCA–Hyperoxia led to 1,599 differentially expressed genes. Hyperoxia without HCA demonstrated upregulated genes associated with inflammatory signaling and immune modulation (including *CXCL8, TM4SF19,* and *PLPP3*) and downregulated genes involved in pathogen recognition and monocyte survival (*TLR6, TLR8,* and *PECAM1).* Sequential HCA–Hyperoxia exposure was associated with a broader transcriptional response, with upregulation of pro-inflammatory and pro-fibrotic genes *(CLEC5A, FN1,* and *CCL24)* and downregulation of immune regulatory genes *(USP18, TMEM176B,* and *HLA-DQB1).* Pathway analysis revealed alterations in immune and inflammatory signaling, antigen presentation, neutrophil degranulation, protein ubiquitination, and metabolic regulation.

**Conclusions:**

Sequential exposure to histological chorioamnionitis and postnatal hyperoxia induces alterations in gene expression and molecular pathways in neonatal cord blood monocytes, affecting immune regulation, which could potentially contribute to the pathogenesis of short- and long-term diseases.

## 1 Introduction

Research on innate immune function in newborns is gaining momentum, with particular emphasis on how immune cells such as monocytes and macrophages respond to various harmful stimuli. Histological chorioamnionitis (HCA), sometimes referred to as intrauterine inflammation, is an inflammatory process that variably affects the chorioamniotic membranes with or without bacterial proliferation. It occurs in approximately 3%–5% of term deliveries, 40%–70% of preterm deliveries, and 94% of very preterm (21–24 weeks) deliveries (1, 2). HCA is known to drive neonatal immune dysregulation, especially in preterm neonates, with persistently abnormal cytokine profiles (3), but similar studies in term infants remain lacking. This fetal immune “activation” or priming has also been associated with change in T cell and neutrophil function, with increased pathogenic T helper cells, decreased regulatory T cells, and amplified neutrophil function (4). HCA has also been shown to have an association with several diseases like cerebral palsy, necrotizing enterocolitis, chronic lung disease, and early childhood asthma, many of which are associated with altered immune function (5–9). In addition, fetal adversity is well described to be a risk factor for early-onset cardiovascular and metabolic diseases in adulthood (Barker hypothesis). The molecular mechanisms of these long-term disease outcomes are not well understood, but immune dysfunction and possible immune reprogramming may be involved in these Developmental Origins of Health and Disease pathways (10, 11).

Hyperoxia is another noxious exposure that has been shown to alter innate immune responses in preterm and term neonates. For example, 65% oxygen priming of neonatal cord blood monocyte-derived macrophages followed by lipopolysaccharide (LPS) treatment resulted in an exaggerated pro-inflammatory macrophage response, with increased cytokine release and a sustained pro-inflammatory transcriptomic profile, potentially mediated in part by downregulation of early growth response 2 (Egr2) and growth factor independence 1 (Gfi1) (12). In a similar study on murine lung macrophages using a double-hit model of hyperoxia followed by LPS, the authors showed that there was an exaggerated immune response associated with a decrease in anti-inflammatory homeostatic macrophages and greater Siglec-F intermediate population of macrophages in lungs (13). In another murine model using global transcriptomic profiling in prenatal chorioamnionitis followed by postnatal hyperoxia, Shrestha et al. demonstrated upregulation of chemokine-mediated signaling and immune cell chemotaxis genes in resident pulmonary immune cells (14).

Global gene expression profiling using microarray is widely employed to discover pathogenic molecular pathways following stimulus exposure. Transcriptome analysis can help uncover changes in gene signatures and find molecular targets to use in the diagnosis and treatment of various disease processes. To date, not much is known about the global gene expression of cord blood monocytes in models of neonatal inflammation. We previously demonstrated 388 genes differentially expressed in neonatal cord blood monocytes exposed to HCA, with key genes regulating inflammatory, immune, respiratory and neurological pathways (15). In this study, we designed a model to explore the changes in neonatal cord blood monocytes following sequential exposure to intrauterine inflammation due to maternal HCA and subsequent hyperoxia *in vitro*. We hypothesize that this double-hit model will result in altered gene expression, which may mediate the development of some of the short- and long-term disease phenotypes seen in survivors of neonatal disease.

## 2 Materials and methods

### 2.1 Study design

The Institutional Review Board (IRB) of Thomas Jefferson University Hospital and the Nemours Institutional Biosafety Committee approved the protocol and procedures in this study. Informed consent was waived by the IRB as this study was performed on discarded blood and placental tissue samples, and no clinical or protected health information was used. This pilot prospective descriptive observational study examined global gene expression in cord blood monocytes of term infants born to mothers with and without HCA, followed by hyperoxia exposure *in vitro*. We excluded patients with maternal infections other than HCA (including COVID-19), severe growth restriction, and major congenital/chromosomal anomalies.

### 2.2 Sample collection and processing

#### 2.2.1 Placental tissues collection

Soon after delivery, the umbilical cord was cut and the placenta was delivered. The placenta, umbilical cord, and placental membranes were grossly examined, and samples were submitted for histopathology, as previously described. A blinded pathologist (JC) then classified the specimens as either HCA (placental membranes score ≥ stage 1) or no HCA (no histological inflammatory changes in fetal membranes), as described in our previous study (15).

#### 2.2.2 Umbilical cord blood collection and monocyte isolation

Approximately 30–40 mL of free flowing cord blood was decanted from the placental end of the umbilical cord into 50 mL polypropylene tubes containing 5 mL of 0.15M ethylenediaminetetraacetic acid (EDTA) in phosphate-buffered saline (PBS). Tubes were gently inverted a few times and then processed for monocyte isolation within 2 h. We prepared a Ficoll-Paque Plus density gradient (GE Healthcare Biosciences, Piscataway, NJ, USA) to separate monocytes from erythrocytes and leucocytes using the manufacturer's guidelines and previously described methodology (15, 16). After Ficoll-Paque Plus density gradient centrifugation, we carefully collected the band of cells with surrounding plasma and washed with EDTA/PBS. This cell pellet was reconstituted again in clear plasma and EDTA/PBS and platelets were removed by centrifugation. Monocytes were then isolated using a Percoll Plus density gradient centrifugation (GE Healthcare Inc., Chicago, IL, USA) as per the manufacturer's guidelines. For monocyte culture, cells were resuspended in sterile Dulbecco's modified eagle medium (MilliporeSigma, Burlington, MA, USA) containing 10% heat-inactivated fetal bovine serum (MilliporeSigma, Burlington, MA, USA) supplemented with penicillin-streptomycin (ATCC, Manassas, VA, USA) and L-Glutamine (MilliporeSigma, Burlington, MA, USA). Approximately 2 × 10⁶ cells were plated in each well of a six-well plate. Monocyte viability was assessed using trypan blue exclusion staining, which showed that approximately 95% of the cells were viable. Monocytes from both the HCA (*n* = 7) and no HCA (*n* = 7) groups were incubated for 18–20 h in either (A) room air (21% FiO_2_) or (B) 95% FiO_2_ in the hyperoxia chamber to yield a total of 28 samples (*n* = 7 for each of the 4 conditions described in the following). We used 95% FiO_2_ to maximize oxygen exposure. The cells were then retrieved and pellets were saved at −80°C for future RNA extraction. Gene expression profiling of monocytes was performed in four groups (1): no HCA–Normoxia (control), (2) HCA–Normoxia, (3) no HCA–Hyperoxia, and (4) HCA–hyperoxia.

### 2.3 RNA isolation and microarray analysis

We isolated RNA from the monocyte cells after hyperoxia or normoxia exposure using the Qiagen miRNeasy Mini Kit (Qiagen, Germantown, MD, USA), as previously described (15). RNA was then quantified on a Nanodrop ND-2000 spectrophotometer (Thermo Fisher Scientific, Waltham, MA, USA). To assess quality, we used Agilent 2200 TapeStation (Agilent Technologies, Santa Clara, CA, USA), as previously described (15). All arrays presented in this study passed quality control. Global gene expression was determined using Affymetrix human Clariom-S chips. To minimize technical batch effects, all RNA samples were processed simultaneously in a single experimental batch. Labeling, hybridization, washing, staining, scanning, and CEL file generation were performed under identical experimental conditions using the same reagents, equipment, and protocol. Consequently, no laboratory processing batches were introduced into the dataset. Raw CEL files were analyzed together in transcriptome analysis console (TAC) software using the single space transformation-robust multiarray average (SST-RMA) workflow, including background correction, quantile normalization, and probe summarization.

### 2.4 Statistical analysis

Global gene expression was analyzed using TAC software version 4.0, as described earlier. Significant differentially expressed genes were identified using a fold-change threshold of ≥1.5 and a *p*-value cutoff of ≤0.05. These genes were subsequently analyzed using ingenuity pathway analysis (IPA; Winter Release, December 2025; QIAGEN Inc.; https://www.qiagenbioinformatics.com/products/ingenuitypathway-analysis) between January and March 2026 to identify enriched biological pathways and functional networks (17).

## 3 Results

We compared transcriptome signatures between (A) HCA–Normoxia and No HCA–Normoxia (control), (B) No HCA–Hyperoxia and No HCA–Normoxia (control), and (C) HCA–Hyperoxia and No HCA–Normoxia (control).

### 3.1 Differential gene expression

Comparison of HCA–Normoxia and No HCA–Normoxia (control) showed 1225 differentially expressed probe IDs (1,195 genes), of which 758 were upregulated and 467 were downregulated (fold change ≥1.5 *p*-value ≤ 0.05). When No HCA–Hyperoxia monocytes were compared with No HCA–Normoxia (control) monocytes, a total of 546 probe IDs (533 genes) were differentially expressed, of which 192 were upregulated and 354 were downregulated. In contrast, 1,646 differentially expressed probe IDs (1,599 altered genes) were identified after sequential exposure to HCA–Hyperoxia compared with No HCA–Normoxia (control), of which 1,091 were upregulated and 555 were downregulated.

The top 10 upregulated and downregulated genes are shown in Tables 1A–C. For genes upregulated in HCA–Normoxia compared with No HCA–Normoxia, *CLEC5A* (C-type lectin domain family 5, member A), *FN1* (fibronectin 1), *CD101* (cluster of differentiation 101), *FCGR3A* [Fc gamma receptor IIIA (CD16A)], *FCGR2B* [Fc gamma receptor IIB (CD32B)], and *PRAM1* (PML-RARA regulated adaptor molecule 1) were relevant to monocyte function. *IL7R* (interleukin-7 receptor), *IL1RN* (interleukin-1 receptor antagonist), and *HLA-DQB1* (human leukocyte antigen-DQ beta 1) were downregulated in HCA–Normoxia and also played a role in monocyte function. Important genes involved in immune function that were upregulated in the No HCA–Hyperoxia group were *TM4SF19* (transmembrane 4 L six family member 19), *CXCL8* (C-X-C motif ligand 8 also known as Interleukin 8), and *PLPP3* (phospholipid phosphatase 3), whereas *CLEC5A*, *FN1*, *DAB2* [Dab, mitogen-responsive phosphoprotein homolog 2 (Drosophila)], and *CCL24* (C-C motif chemokine ligand 24) were upregulated in the double-hit HCA–Hyperoxia group. Important immune-related genes downregulated in the No HCA–Hyperoxia group are *TLR8* (Toll like receptor 8), *PECAM1* (platelet endothelial cell adhesion molecule-1), *TNFSF10* [tumor necrosis factor (ligand) superfamily member 10], *FPR3* (formyl peptide receptor 3), and *TLR6* (toll like receptor 6) whereas *TMEM176B* (transmembrane protein 176B), *IFIT1* (interferon-induced protein with tetratricopeptide repeats 1), *IFIT2* (interferon-induced protein with tetratricopeptide repeats 2), *USP18* (ubiquitin specific peptidase 18), *OAS3* (2–5-oligoadenylate synthetase 3), *HLA-DQB1* (human leukocyte antigen-DQ beta 1 or major histocompatibility complex, class II, DQ beta 1), and *TNFSF10* (tumor necrosis factor superfamily member 10) were downregulated in the HCA–Hyperoxia group.

**Table 1:** Top 10 upregulated and downregulated genes in 1A. HCA–Normoxia compared with No HCA–Normoxia (control), 1B. No HCA–Hyperoxia compared with No HCA–Normoxia (control) and 1C. HCA–Hyperoxia compared with No HCA–Normoxia (control).

Upregulated genes				Downregulated genes			
Probe ID	Genes	Fold change	*p*-Value	Probe ID	Genes	Fold change	*p*-Value
1A. HCA–Normoxia compared with No HCA–Normoxia (control)							
TC0700012849.hg.1	*CLEC5A*	32.34	0.0055	TC0200016484.hg.1	*RGPD2; RGPD1*	−7.26	0.0089
TC0200015650.hg.1	*FN1*	22.64	0.0058	TC0600011499.hg.1	*HLA-DQB1*	−5.43	0.0429
TC0100009560.hg.1	*CD101*	10.18	0.0002	TC0900011613.hg.1	*FAM102A*	−4.12	0.0483
TC0100016185.hg.1	*FCGR3A*	8.31	0.001	TC0500007138.hg.1	*IL7R*	−3.92	0.0473
TC0100016189.hg.1	*FCGR3B*	7.81	0.0065	TC0200016511.hg.1	*IL1RN*	−3.75	0.0015
TC0100018310.hg.1	*FCGR2B*	7.75	0.0003	TC0600007868.hg.1	*RNF8*	−3.15	0.0007
TC0500008632.hg.1	*SLC22A5*	6.72	0.0048	TC1600006445.hg.1	*HBA2*	−3.1	0.0312
TC1500006925.hg.1	*THBS1*	6.33	0.0078	TC1600006446.hg.1	*HBA1*	−3.07	0.0433
TC1900009546.hg.1	*PRAM1*	6.22	0.0058	TC1200007209.hg.1	*DDX11*	−3.04	0.0009
TC0400012947.hg.1	*GPRIN3*	5.89	0.0003	TC0600011173.hg.1	*GUSBP2*	−2.95	0.0093
1B. No HCA–Hyperoxia compared with No HCA–Normoxia (control)							
TC0200016648.hg.1	*CDC42EP3*	4.79	0.0106	TC0100014857.hg.1	*GBP5*	−7.25	0.0295
TC0800010607.hg.1	*ASPH*	4.45	0.0037	TC0X00006626.hg.1	*TLR8*	−6.68	0.0164
TC1900009824.hg.1	*DNAJB1*	3.81	0.0013	TC1700011442.hg.1	*PECAM1*	−6.52	0.0089
TC0300014095.hg.1	*TM4SF19*	3.48	0.0031	TC0300013146.hg.1	*TNFSF10*	−6.37	0.0426
TC0400007836.hg.1	*CXCL8*	2.96	0.0033	TC1700012440.hg.1	*RNFT1*	−4.93	0.0104
TC0300014092.hg.1	*TM4SF19-TCTEX1D2*	2.85	0.0009	TC0700011584.hg.1	*FGL2*	−4.83	0.0371
TC0100014313.hg.1	*PPAP2B*	2.84	0.0308	TC1200010618.hg.1	*TUBA1A*	−4.59	0.0456
TC0100018443.hg.1	*PLPP3*	2.8	0.026	TC1900008669.hg.1	*FPR3*	−4.48	0.0455
TC1100010054.hg.1	*NRIP3*	2.69	0.0489	TC0400012922.hg.1	*TLR6*	−4.39	0.0428
TC0500008054.hg.1	*POLR3G*	2.69	0.0085	TC0300010358.hg.1	*XPC*	−4.33	0.0063
1C. HCA–Hyperoxia compared with No HCA–Normoxia (control)							
TC0700012849.hg.1	*CLEC5A*	79.2	8.53E^−05^	TC0700013047.hg.1	*TMEM176B*	−20.54	0.0476
TC0200015650.hg.1	*FN1*	28.26	0.0082	TC1000008400.hg.1	*IFIT1*	−10.59	0.0264
TC2100008027.hg.1	*KCNE1*	11.65	0.0133	TC2200006540.hg.1	*USP18*	−9.31	0.0196
TC0500013301.hg.1	*DAB2*	10.15	0.0075	TC1000008396.hg.1	*IFIT2*	−6.27	0.0238
TC1300006979.hg.1	*RGCC*	9.78	1.93E^−05^	TC0100010874.hg.1	*RGL1*	−6.11	0.0095
TC0400012947.hg.1	*GPRIN3*	9.33	3.66E^−05^	TC0200012652.hg.1	*PNPT1*	−5.61	0.0365
TC0800008299.hg.1	*SDC2*	8.96	0.0008	TC0600011499.hg.1	*HLA-DQB1*	−5.51	0.0369
TC0700011561.hg.1	*CCL24*	8.94	0.0408	TC0200016484.hg.1	*RGPD2; RGPD1*	−5.24	0.0088
TC0600009381.hg.1	*CENPW*	8.86	0.0001	TC1200008920.hg.1	*OAS3*	−4.49	0.0431
TC0500007895.hg.1	*CMYA5*	8.57	0.0058	TC0300013146.hg.1	*TNFSF10*	−4.26	0.0412

Figure 1 presents a Venn diagram illustrating the overlap of differentially expressed genes among the three comparison groups. A total of 36 genes were identified as altered across all three groups, as shown in Supplementary Table 1.

**Figure 1:**
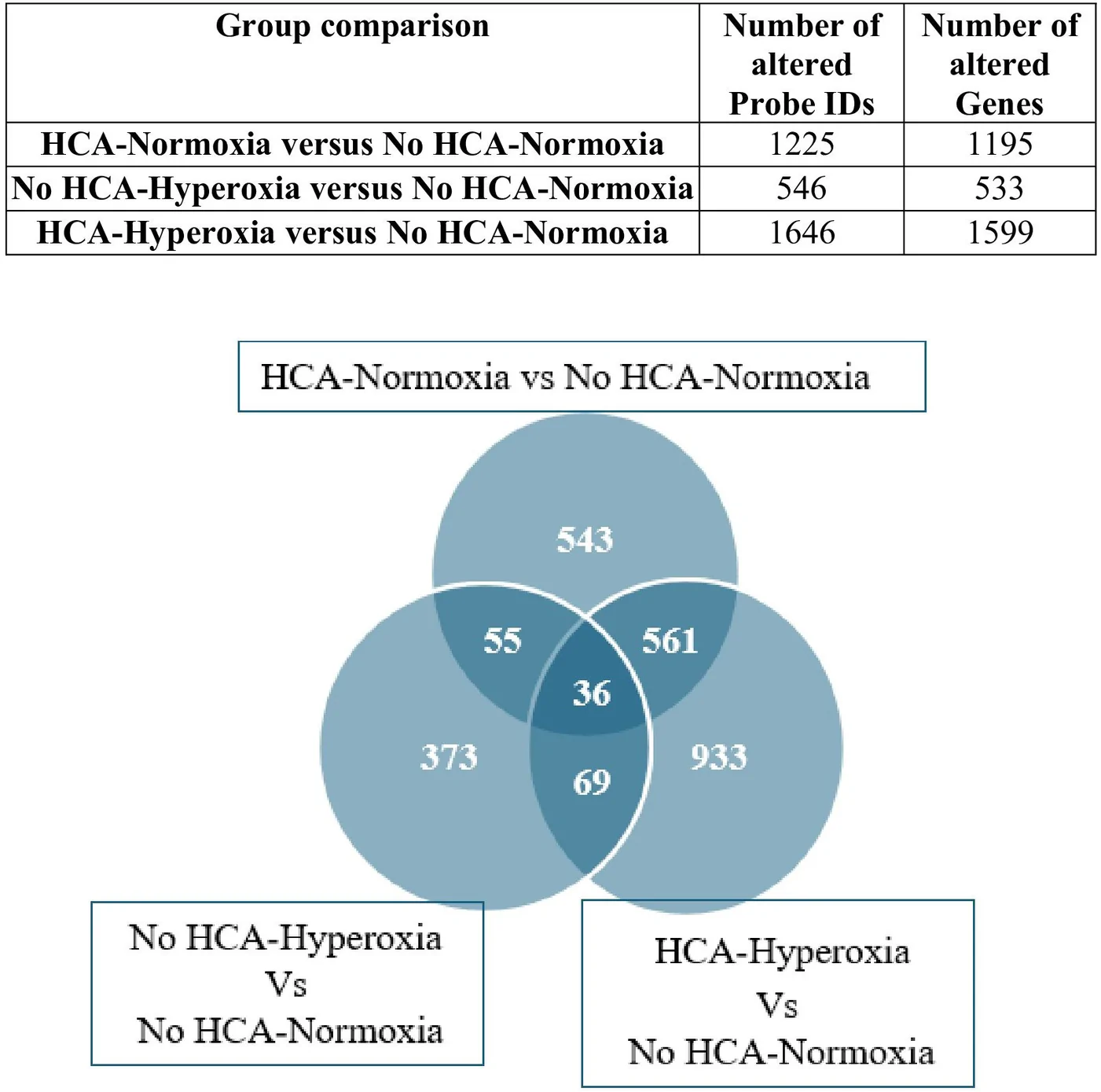
Venn diagram showing overlap of gene transcription signatures (fold change ≥1.5 and *p*-value ≤0.05) between the three comparison groups.

### 3.2 Ingenuity pathway analysis

Monocytes in the HCA–Normoxia group showed 397 significantly altered canonical pathways (*p* ≤ 0.05), whereas oxygen exposure alone (No HCA–Hyperoxia) showed 133 significantly altered canonical pathways. Monocytes in the HCA–Hyperoxia group showed 480 significantly modified canonical pathways when compared with No HCA–Normoxia. Table 2 shows the top five canonical pathways in the three groups. Compared with the No HCA–Normoxia control group, the HCA–Normoxia group exhibited significant alterations in several key canonical pathways, including neutrophil degranulation, mitochondrial dysfunction, oxidative phosphorylation, respiratory electron transport, and class I MHC-mediated antigen processing and presentation. In the No HCA–Hyperoxia group compared with the No HCA–Normoxia (control) group, the most important canonical pathways included SUMOylation of DNA damage response and repair proteins, the sirtuin signaling pathway, FXR/RXR activation, retinol biosynthesis, and TREM-1 signaling. Neutrophil degranulation, Class I MHC-mediated antigen processing and presentation, the protein ubiquitination pathway, the RHO GTPase cycle, and activation of gene expression by SREBF (SREBP) were the pathways implicated in the HCA–Hyperoxia monocytes compared with the No HCA–Normoxia (control) group. The most significantly altered disease and disorder categories in the HCA–Normoxia group relative to controls included cancer, organismal injury and abnormalities, gastrointestinal disease, endocrine system disorders, and dermatological diseases and conditions. Important modified diseases and functions in the No HCA–Hyperoxia group included cancer, organismal injury and abnormalities, endocrine system disorders, gastrointestinal disease, and dermatological diseases and conditions. In the HCA–Hyperoxia group, modified diseases included organismal injury and abnormalities, cell death and survival, infectious diseases, cellular development, cellular growth and proliferation, immunological disease, and inflammatory diseases (Table 3).

**Table 2:** Top canonical pathways from ingenuity pathway analysis.

Group comparison	Name	*p*-Value	Number of genes involved	Molecules involved in pathways
HCA–Normoxia versus No HCA–Normoxia (control)	Neutrophil degranulation	1.23E^−15^	62	ACTR1B,ADGRE5,ALOX5,ARSA,ARSB,BST2,CAB39,CAP1,CAT,CD36,CD44,CD53,CEACAM1,CLEC4D,CLEC5A,COTL1,CPPED1,CRISPLD2,CTSS,CXCR1,CYBA,DYNC1LI1,FCGR2A,FCGR3B,FGL2,GCA,GGH,GRN,GSTP1,HPSE,HSPA6,IQGAP2,ITGAV,ITGAX,ITGB2,KCNAB2,LAMP1,LAMTOR3,LTA4H,MAN2B1,OLR1,PECAM1,PGLYRP1,PRDX4,PSAP,PSMA5,PSMD14,QSOX1,RAC1,RHOA,RHOG,RNASE3,SERPINA1,SIRPB1,SLC11A1,STK10,STOM,SVIP,TLR2,TMT1A,TXNDC5,VAT1
	Mitochondrial dysfunction	2.84E^−10^	42	ACADL,ACO2,APH1A,APP,ATP1B1,ATP5MC3,ATP5ME,ATP5MGL,ATP5PB,ATP5PO,BID,CAMK2D,COX5A,COX6B1,COX7A1,COX7A2,COX8A,COXFA4,CYCS,DLD,FIS1,GPD2,GPX3,GSTP1,ITPR1,LRRK2,MCU,MYH9,NDUFA13,NDUFA6,NDUFAB1,NDUFB5,NDUFB9,NDUFV3,PIK3C2B,PIK3R6,PPARG,PRKACA,PRKN,SDHD,UQCRC1,UQCRFS1
	Oxidative phosphorylation	1.29E^−09^	21	ATP5MC3,ATP5ME,ATP5MGL,ATP5PB,ATP5PO,COX5A,COX6B1,COX7A1,COX7A2,COX8A,COXFA4,CYCS,NDUFA13,NDUFA6,NDUFAB1,NDUFB5,NDUFB9,NDUFV3,SDHD,UQCRC1,UQCRFS1
	Respiratory electron transport	1.88E^−08^	18	COX5A,COX6B1,COX7A1,COX7A2,COX8A,COXFA4,CYCS,ETFB,ETFDH,NDUFA13,NDUFA6,NDUFAB1,NDUFB5,NDUFB9,NDUFV3,SDHD,UQCRC1,UQCRFS1
	Class I MHC mediated antigen processing and presentation	7.01E^−08^	40	ANAPC11,ASB8,BCAP31,CD36,CTSS,CUL1,CYBA,FBXL4,FBXO31,ITGAV,KLHL2,KLHL20,KLHL21,KLHL22,LNPEP,NCF2,PJA1,PRKN,PSMA5,PSMA6,PSMB10,PSMB2,PSMB4,PSMB8,PSMB9,PSMD14,PSMD5,RBX1,RNF217,SEC13,SEC61B,SH3RF1,TLR1,TLR2,TLR6,TRIM69,UBA6,UBE2C,UBE2J2,UBE2W
No HCA–Hyperoxia versus No HCA–Normoxia (control)	SUMOylation of DNA damage response and repair proteins	8.77E^−05^	8	EID3, NUP85, PHC2, RAE1, SMC1A, TPR, XPC, XRCC4
	Sirtuin signaling pathway	1.19E^−04^	16	AGTRAP, ATG16L1, ATP5F1C, CXCL8, GADD45B, GTF3C2,IDH2, NDUFA10, NDUFA11, NDUFB10, PPARG, TIMM23, TUBA1A, UCP2, XPA,XPC
	FXR/RXR activation	2.57E^−04^	12	CNTF, CXCL8, DDIT3, GCLM, GSTK1, IFNG, IL36RN,MAPK9, NLRP3, PPARG, PRKAB1, TXNIP
	Retinol biosynthesis	4.36E^−04^	6	AADAC, CES2, CES5A, DAGLB, DHRS7, RDH10
	TREM-1 signaling	5.96E^−04^	7	CD83, CD86, CXCL8, ITGAX, NLRP3, TLR6, TLR8
HCA–Hyperoxia versus No HCA–Normoxia (control)	Neutrophil degranulation	3.14E^−15^	73	ACP3,APEH,ARSA,ARSB,CAP1,CD36,CD44,CD58,CEACAM1,CLEC4D,CLEC5A,COTL1,CPNE3,CRISPLD2,CYBA,DEGS1,DNAJC3,DOCK2,DOK3,DYNC1LI1,FCAR,FCGR2A,FCGR3B,GGH,GLA,GM2,GMFG,GOLGA7,GPR84,GSTP1,HGSNAT,HPSE,HSPA6,IMPDH2,IQGAP2,ITGAV,ITGAX,KCNAB2,LAMP1,LAMTOR3,LGALS3,LTA4H,MAN2B1,MCEMP1,MGAM,MGST1,MMP8,MOSPD2,OLR1,P2RX1,PGLYRP1,PGM1,PLAUR,PLEKHO2,PRDX4,PSAP,PSMA5,PSMB1,PSMD7,PTPN6,QSOX1,RAB7A,RAC1,RAP2B,RHOA,RHOG,S100A11,SDCBP,SIRPA,SIRPB1,SLC11A1,TLR2,TOM1
	Class I MHC mediated antigen processing and presentation	2.87E^−12^	58	ANAPC11,ANAPC7,AREL1,ASB8,BCAP31,BLMH,BTRC,CALR,CD3,CHUK,CUL5,CYBA,ELOC,FBXO21,FBXW7,HSPA5,ITGAV,KEAP1,KLHL2,KLHL20,LONRF1,MRC2,NCF2,PSMA3,PSMA5,PSMA6,PSMB1,PSMB10,PSMB4,PSMB5,PSMB8,PSMC6,PSMD4,PSMD7,PSMD8,RBX1,RNF114,RNF41,SEC13,SEC22B,SEC61B,SH3RF1,SPSB1,TLR2,TLR4,TLR6,TRIM69,UBC,UBE2C,UBE2J1,UBE2J2,UBE2 K,UBE2Q2,UBE2R2,UBE2V1,UBE2W,UBE2Z,UNKL
	Protein Ubiquitination Pathway	6.56E^−12^	47	ANAPC11,BAP1,BTRC,CBL,DNAJB11,DNAJB12,DNAJB5,DNAJC21,DNAJC28,DNAJC3,DNAJC7,ELOC,FBXW7,HSCB,HSPA5,HSPA6,HSPD1,MDM2,PSMA3,PSMA5,PSMA6,PSMB1,PSMB10,PSMB4,PSMB5,PSMB8,PSMC6,PSMD4,PSMD7,PSMD8,RBX1,SUGT1,UBC,UBE2C,UBE2I,UBE2J1,UBE2J2,UBE2 K,UBE2Q2,UBE2R2,UBE2V1,UBE2W,UBE2Z,USO1,USP18,USP2,USP32
	RHO GTPase cycle	2.76E^−10^	60	ABI1,ABR,ACTG1,ADD3,ALDH3A2,ARAP1,ARFGAP3,ARHGAP11B,ARHGAP18,ARHGAP24,ARHGAP26,ARHGEF2,ARHGEF6,BAIAP2,BCAP31,BLTP3B,CCT6A,CDC42EP3,COPS4,CPD,CYBA,DBT,DNMBP,DOCK1,DOCK10,DOCK2,DOCK5,DOCK7,EMD,FARP2,FGD3,FGD4,FMNL3,GIT1,GOLGA8 K (includes others), IQGAP2,LCK,MOSPD2,MTX1,MYO6, MYO9B,NCF2,RAB7A, RAC1,RAPGEF1,RHOA,RHOG,RHOQ, SH3RF1,SHKBP1,SOWAHC,TAOK3,TEX2,TFRC,TRIO,VIM,WASL,WDR11,WDR6,WDR91
	Activation of gene expression by SREBF (SREBP)	3.05E^−10^	16	ACACA,CYP51A1,DHCR7,FDFT1,FDPS,GGPS1,HELZ2,HMGCR,IDI1,LSS,MTF1,NCOA6,SC5D,SCD,SQLE,SREBF2

**Table 3:** Top modified diseases and functions from ingenuity pathway analysis.

Group comparison	Modified disease and functions	Number of genes involved	Range of *p*-value for genes involved
HCA–Normoxia versus No HCA–Normoxia (control)	Cancer	1,109	1.52E^−118–^4.15E^−06^
	Organismal injury and abnormalities	1,117	1.52E^−118–^4.74E^−06^
	Gastrointestinal disease	1,057	1.24E^−83–^3.85E^−06^
	Endocrine system disorders	1,004	1.95E^−70–^2.02E^−06^
	Dermatological diseases and conditions	860	5.03E^−54–^3.45E^−06^
	Neurological disease	901	2.88E^−41–^3.97E^−06^
	Reproductive system disease	809	1.65E^−38–^3.85E^−06^
	Hepatic system disease	659	1.89E^−32–^3.56E^−06^
	Cellular function and maintenance	697	2.85E^−30–^3.92E^−06^
	Protein synthesis	582	1.87E^−28–^3.63E^−06^
No HCA–Nyperoxia versus No HCA–Normoxia (control)	Cancer	482	1.26E^−48–^7.55E^−03^
	Organismal injury and abnormalities	486	1.26E^−48–^7.98E^−03^
	Endocrine system disorders	429	2.4E^−32–^5.49E^−03^
	Gastrointestinal disease	448	4.48E^−32–^7.47E^−03^
	Dermatological diseases and conditions	352	6.8E^−16–^7.86E^−03^
	Reproductive system disease	354	7.8E^−16–^6.34E^−03^
	Neurological disease	378	1.95E^−15–^7.86E^−03^
	Hepatic system disease	284	3.36E^−15–^6.25E^−03^
	Renal and urological disease	238	1.27E^−09–^7.98E^−03^
	Hereditary disorder	174	1.55E^−08–^7.05E^−03^
HCA–Hyperoxia versus No HCA–Normoxia (control)	Organismal injury and abnormalities	1,513	1.03E^−43–^2.43E^−03^
	Cell death and survival	494	5.52E^−14–^2.43E^−03^
	Infectious diseases	358	3.34E^−13–^2.34E^−03^
	Cellular development	460	3.92E^−13–^2.34E^−03^
	Cellular growth and proliferation	459	3.92E^−13–^2.25E^−03^
	Immunological disease	656	4.26E^−13–^2.34E^−03^
	Inflammatory disease	335	4.26E^−13–^2.34E^−03^
	Cellular function and maintenance	478	1.28E^−11–^2.36E^−03^
	Inflammatory response	329	1.28E^−11–^2.34E^−03^
	Cell-to-cell signaling and interaction	270	2.86E^−11–^2.18E^−03^

Analysis of upstream regulators revealed that the top predicted upstream regulators in the HCA–Normoxia group relative to controls were IFNG (interferon gamma), dexamethasone (medication), immunoglobulin (complex), and lipopolysaccharide (endotoxin), with predicted activation of all except immunoglobulin. TREM-1 (Triggering Receptor Expressed on Myeloid cells (1), FAS (first apoptosis signal receptor or Fas cell surface death receptor), dexamethasone, GSK-J4 (small-molecule chemical inhibitor), and MAP2K4 (mitogen-activated protein kinase kinase 4) were involved in the hyperoxia group, of which TREM-1 and GSK-J4 were predicted to be activated compared with the No HCA–Normoxia (control) group. In the HCA–Hyperoxia group compared with the No HCA–Normoxia group, HNF4A (hepatocyte nuclear factor 4 alpha), lipopolysaccharide, arsenic trioxide, TNF (tumor necrosis factor), IFNG, and dexamethasone were the important upstream regulators involved, with predicted activation of all except HNF4A (Table 4).

**Table 4:** Top upstream regulators from ingenuity pathway analysis.

Group comparison	Upstream regulator	Molecule type	Predicted activation state	Activation z-score	*p*-Value of overlap
HCA–Normoxia versus No HCA–Normoxia (control)	IFNG	Cytokine	Activated	2.73	8.57E^−21^
	dexamethasone	Chemical drug	Activated	2.161	7.13E^−18^
	IMMUNOGLOBULIN (complex)	Complex	Inhibited	−2.315	1.12E^−16^
	lipopolysaccharide	Chemical drug	Activated	5.688	3.19E^−16^
	HNF4A	Transcription regulator		−0.104	1.37E^−15^
	TNF	Cytokine	Activated	3.909	1.85E^−12^
	Camptothecin	Chemical drug		1.147	4.94E^−12^
	CSF1	Cytokine	Activated	2.515	5.01E^−12^
	TP73	Transcription regulator		1.226	5.11E^−12^
	TP53	Transcription regulator	Activated	2.026	6.25E^−12^
No HCA–Hyperoxia versus No HCA–Normoxia (control)	TREM-1	Transmembrane receptor	Activated	2.665	1.54E^−11^
	FAS	Transmembrane receptor		−0.798	1.22E^−07^
	dexamethasone	Chemical drug		−1.126	3.61E^−06^
	GSKJ4	Chemical reagent	Activated	2.538	4.75E^−06^
	MAP2K4	Kinase		0.104	7.42E^−06^
	HNF4A	Transcription regulator		−1.373	7.43E^−06^
	TNFRSF9	Transmembrane receptor		0.249	1.16E^−05^
	GW9662	Chemical reagent		−0.523	0.000018
	Ascomycin	Chemical reagent			2.23E^−05^
	Camptothecin	Chemical drug	Activated	2.375	2.31E^−05^
HCA–Hyperoxia versus No HCA–Normoxia (control)	HNF4A	transcription regulator		−0.327	1.36E^−26^
	lipopolysaccharide	Chemical drug	Activated	6.114	2.42E^−25^
	arsenic trioxide	Chemical drug	Activated	2.612	1.08E^−24^
	TNF	Cytokine	Activated	5.768	4.23E^−22^
	IFNG	Cytokine	Activated	2.065	4.72E^−22^
	Dexamethasone	Chemical drug	Activated	2.532	2.85E^−21^
	beta-estradiol	Chemical—endogenous mammalian	Activated	2.882	2.94E^−21^
	TGFB1	Growth factor	Activated	3.557	4.11E^−21^
	LDL (complex)	Complex	Activated	2.765	4.88E^−21^
	Immunoglobulin (complex)	Complex		−1.068	1.61E^−19^

Tables 5A–C present the top toxicological functions implicated in HCA–Normoxia, No HCA–Hyperoxia, and HCA–Hyperoxia when compared with No HCA–Normoxia, respectively.

**Table 5:** IPA results for Toxicological analysis.

Organ system	Name	*P*-value range	Number of molecules involved
A. Top toxicological functions with HCA–Normoxia compared with No HCA–Normoxia (control)			
Cardiotoxicity	Cardiac enlargement	1–1.99E^−05^	76
	Cardiac infarction	1– 2.67E^−05^	53
	Cardiac dysfunction	5.86E^−01–^4.49E^−05^	59
	Cardiac arteriopathy	4.67E^−01–^1.26E^−04^	72
	Cardiac necrosis/cell death	4.67E^−01–^1.15E^−03^	30
Hepatotoxicity	Liver hyperplasia/hyperproliferation	1–2.47E^−27^	575
	Liver necrosis/cell death	3.05E^−01–^6.35E^−11^	48
	Hepatocellular carcinoma	1– 1.02E^−09^	231
	Liver steatosis	2.59E^−01–^2.88E^−09^	78
	Liver damage	1–4.16E^−09^	47
Nephrotoxicity	Renal necrosis/cell death	1–7.48E^−10^	68
	Renal damage	2.28E^−01–^1.18E^−05^	42
	Renal inflammation	5.68E^−01–^9.00E−^05^	49
	Renal nephritis	5.68E^−01–^9.00E^−05^	48
	Nephrosis	1.24E^−01–^1.71E^−04^	21
B. Top toxicological functions with no HCA–Hyperoxia compared with No HCA–Normoxia (control)			
Cardiotoxicity	Cardiac arteriopathy	3.20E^−01–^4.12E^−04^	33
	Cardiac infarction	3.68E^−01–^1.05E^−03^	25
	Cardiac inflammation	3.68E^−01–^3.27E^−03^	9
	Pulmonary hypertension	1–4.73E^−03^	5
	Cardiac enlargement	1– 3.61E^−02^	23
Hepatotoxicity	Liver hyperplasia/hyperproliferation	5.55E^−01–^8.46E^−14^	254
	Hepatocellular carcinoma	5.55E^−01–^1.24E^−06^	107
	Liver damage	5.12E^−01–^4.40E^−05^	17
	Liver fibrosis	3.39E^−01–^5.42E^−03^	23
	Liver steatosis	1.14E^−01–^6.25E^−03^	28
Nephrotoxicity	Renal damage	5.12E^−01–^1.07E^−04^	22
	Renal proliferation	1.51E^−01–^6.61E^−03^	13
	Renal necrosis/cell death	5.16E^−01–^8.89E^−03^	22
	Renal inflammation	4.79E^−01–^1.31E^−02^	22
	Renal nephritis	4.79E^−01–^1.31E^−02^	22
C. Top toxicological functions with HCA–hyperoxia compared with No HCA–Normoxia (control)			
Cardiotoxicity	Cardiac infarction	1– 2.62E^−11^	84
	Cardiac enlargement	1– 2.08E^−09^	108
	Pulmonary hypertension	3.66E^−01–^8.17E^−08^	42
	Cardiac dysfunction	1– 1.39E^−05^	77
	Cardiac necrosis/cell death	4.95E^−01–^1.40E^−05^	45
Hepatotoxicity	Liver hyperplasia/hyperproliferation	1–1.23E^−31^	753
	Hepatocellular carcinoma	1–3.31E^−16^	323
	Liver steatosis	4.34E^−01–^2.36E^−13^	108
	Liver necrosis/cell death	4.87E^−01–^7.82E^−11^	57
	Liver inflammation/hepatitis	1–1.15E^−10^	87
Nephrotoxicity	Renal necrosis/cell death	5.23E^−01–^9.78E^−13^	94
	Kidney failure	4.34E^−01–^1.81E^−07^	46
	Renal inflammation	5.23E^−01–^3.24E^−07^	75
	Renal nephritis	5.23E^−01–^3.24E^−07^	74
	Glomerular injury	1–4.28E^−07^	97

The top networks implicated in HCA alone, hyperoxia alone, and HCA–Hyperoxia are shown in Table 6 and Figures 2A–C. The most significant IPA networks identified in the HCA–Normoxia group relative to controls were enriched for functions related to developmental and hereditary disorders, neurological disease, molecular transport, cellular assembly and organization, organismal injury and abnormalities, cancer, and reproductive system disease (Figure 2A). Networks involved in No HCA–Hyperoxia included cardiovascular disease, hereditary and metabolic disorders, immunological and inflammatory diseases, cancer, organismal injury and abnormalities, hematological disease, and processes involving post-translational modification, cellular function and maintenance, and protein degradation (Figure 2B). In HCA–Hyperoxia, vitamin and mineral metabolism, molecular transport, small-molecule biochemistry, lipid metabolism, developmental disorders, cardiovascular disease, renal and urological disease, and organismal injury and abnormalities were shown to be involved (Figure 2C).

**Table 6:** Top IPA networks.

Group comparison	ID	Associated network functions	Score
HCA–Normoxia compared with No HCA–Normoxia (control)	1	Developmental disorder, Hereditary disorder, Neurological disease	55
	2	Cellular assembly and organization, hereditary disorder, molecular transport	54
	3	Hereditary disorder, neurological disease, organismal injury and abnormalities	48
	4	Cancer, organismal injury and abnormalities, Reproductive system disease	41
No HCA–Hyperoxia compared with No HCA–Normoxia (control)	1	Cardiovascular disease, hereditary disorder, metabolic disease	54
	2	Hereditary disorder, immunological disease, inflammatory disease	51
	3	Cancer, organismal injury and abnormalities, hematological disease	42
	4	Posttranslational modification, cellular function and maintenance, protein degradation	38
HCA–Hyperoxia compared with No HCA–Normoxia (control)	1	vitamin and mineral metabolism, molecular transport, small molecule biochemistry	49
	2	Developmental disorder, organismal injury and abnormalities, renal and urological disease	44
	3	Lipid metabolism, molecular transport, small molecule biochemistry	43
	4	Cardiovascular disease, organismal injury and abnormalities, developmental disorder	41

**Figure 2:**
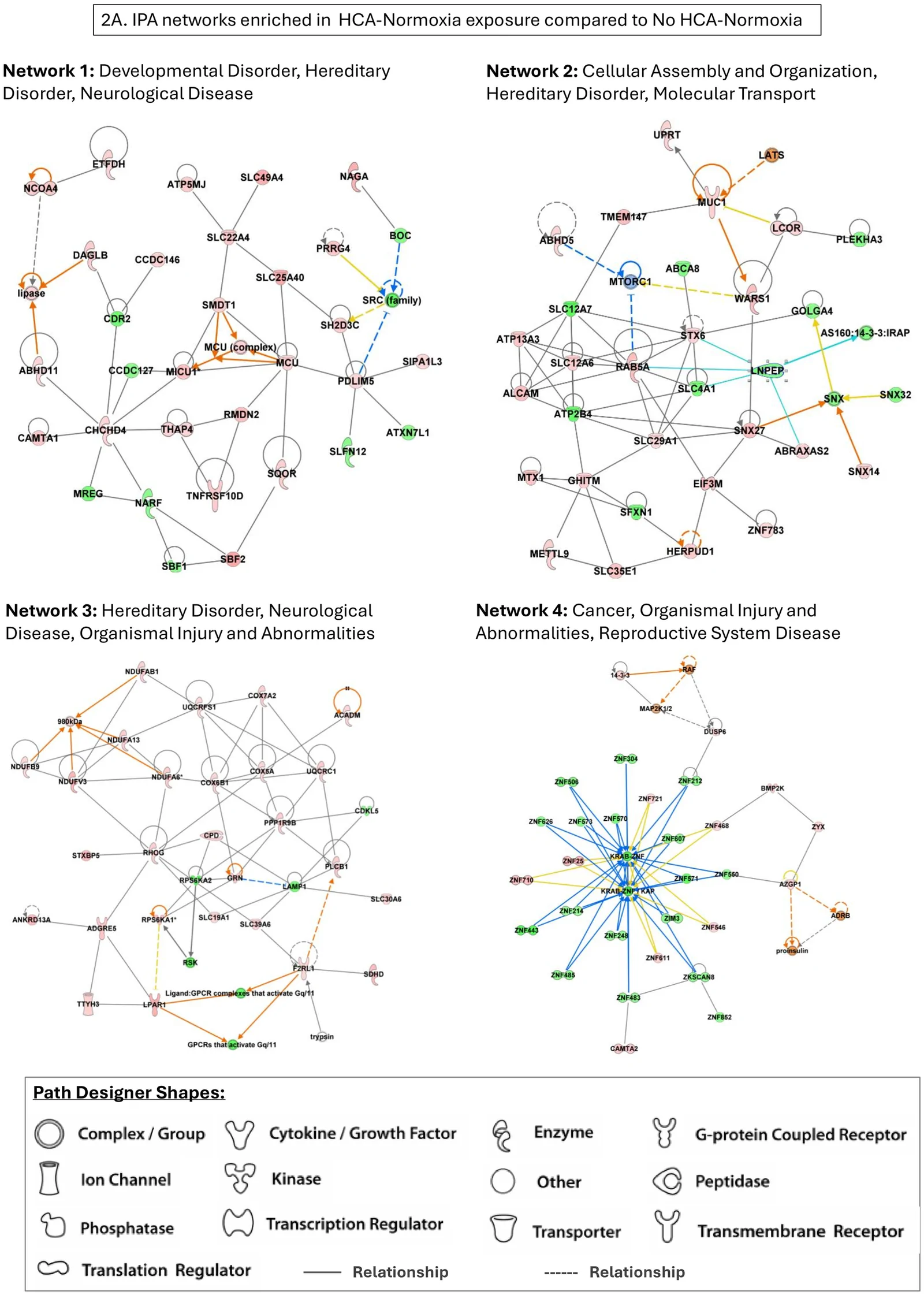

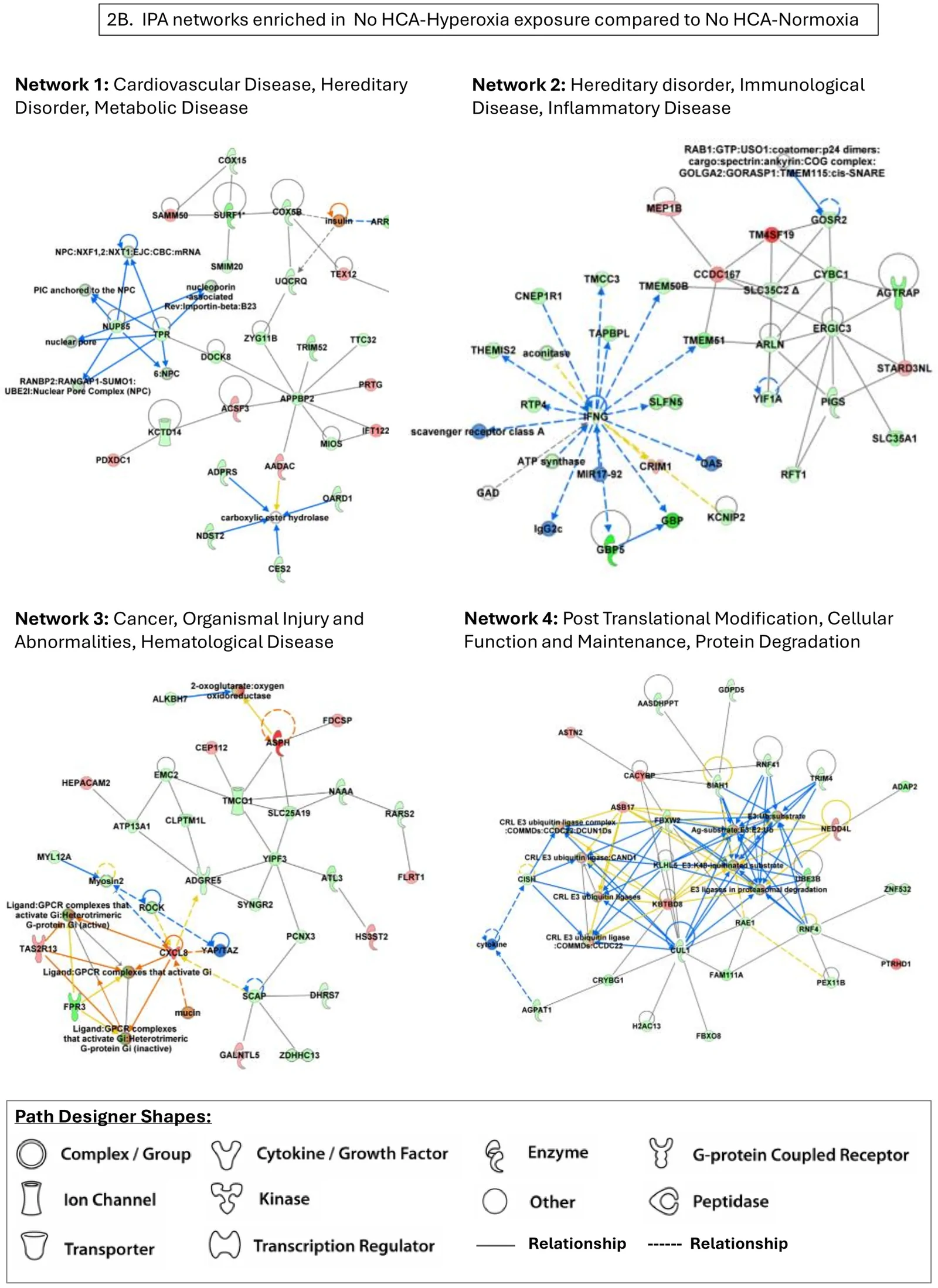

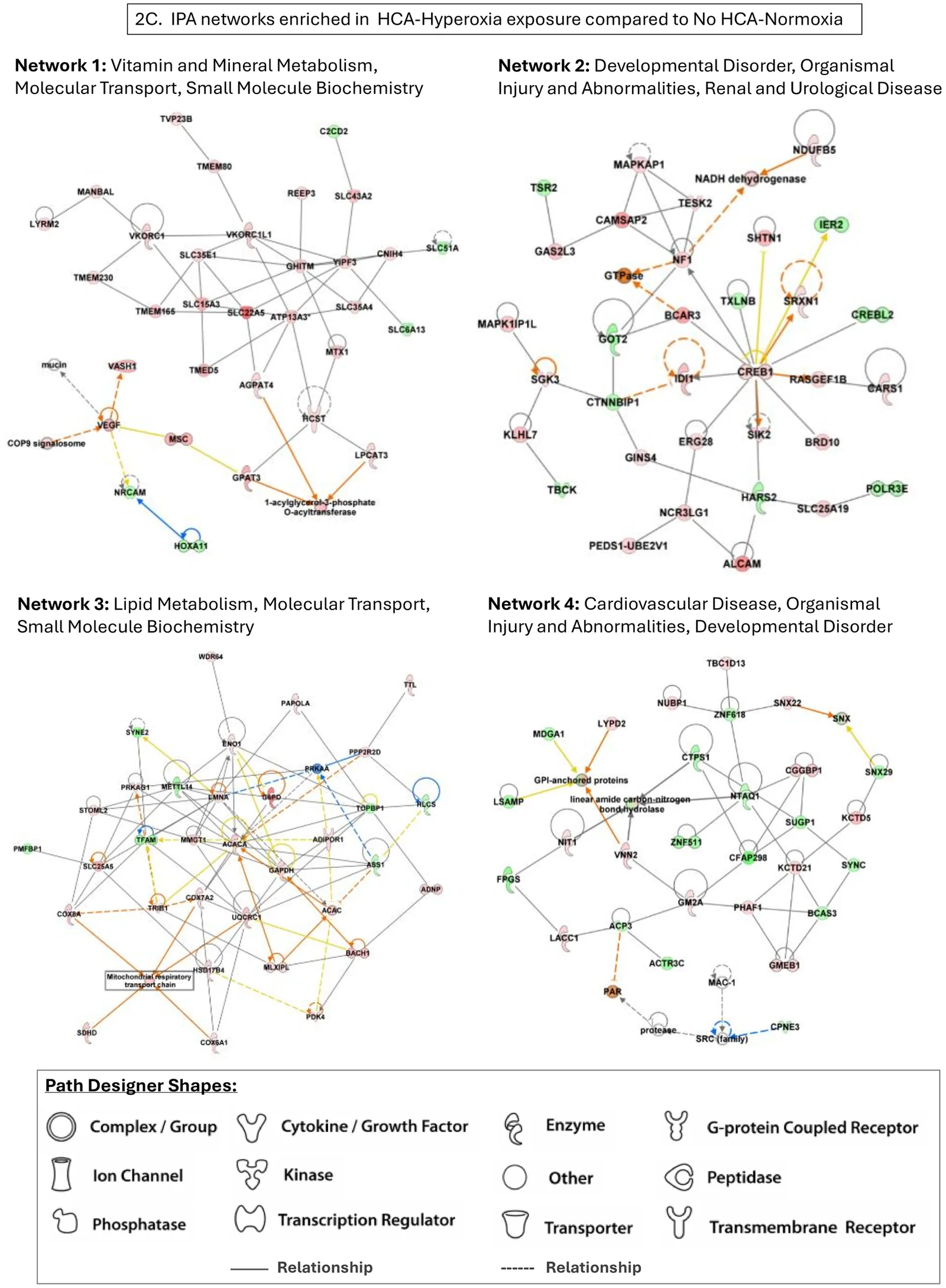
**(A–C)** represent the top four networks identified in each comparison group by ingenuity pathway analysis (IPA) software (QIAGEN Inc., https://www.qiagenbioinformatics.com/products/ingenuitypathway-analysis). **(A)** 1,225 probe sets differentially expressed with HCA–Normoxia exposure compared with the No HCA–Normoxia (control). **(B)** 546 probe sets differentially expressed with No HCA–Hyperoxia exposure compared with the No HCA–Normoxia (control). **(C)** 1,646 probe sets differentially expressed with sequential HCA–Hyperoxia exposure (double-hit) compared with the No HCA–Normoxia (control). Molecules colored red represent upregulation (increased measurement) and molecules in green represent downregulation (decreased measurement). The solid lines denote a direct relationship between the corresponding molecules, whereas the dashed lines indicate that the effect is mediated indirectly via one or more intermediate molecules or mechanisms.

## 4 Discussion

Perinatal inflammation in the form of HCA, followed by hyperoxia exposure, represents two common noxious influences encountered in the newborn period, for both term and preterm neonates. Although both conditions are well known to cause immune dysregulation, this study is the first to describe differential gene expression and altered molecular pathways in monocytes in a double-hit model in term neonates, an area that remains largely unexplored. In human studies, a meta-analysis showed that preterm infants exposed to HCA and oxygen had a higher risk of developing chronic lung disease of prematurity, which is associated with lung inflammation and fibrosis (18). This double-hit model has also been associated with long-term extra-pulmonary sequelae, such as adult left ventricular remodeling and cardiac dysfunction in mice (19), and immune-mediated renal injury (10, 20). The long-term health consequences of preterm birth-related morbidities such as adverse cardiovascular outcomes, obesity, and metabolic syndrome are also well described and could be mediated by functional alterations in various tissues (21). We previously demonstrated altered gene expression in human cord blood monocytes exposed to HCA alone, with key genes modulating immune and infectious pathways affecting long-term lung and neurological disease states (15). The present study elaborates on the alteration in gene expression in human neonate cord blood monocytes after *in vivo* exposure to HCA and subsequent 95% oxygen exposure to explore the role played by altered monocyte gene transcription in this double-hit model. However, larger studies that further characterize these transcriptional changes and clarify their relationship to various disease processes are needed to establish the role of monocytes in disease pathogenesis and identify potential molecular targets for disease prevention and treatment.

Among genes upregulated in HCA–Normoxia compared with No HCA–Normoxia (Table 1A), the prominent upregulation of *CLEC5A*, *FCGR3A*, *FCGR2B*, *CD101*, and *PRAM1* could suggest activation and differentiation of monocyte populations in HCA, while increased *FN1* expression is consistent with monocyte/macrophage-mediated extracellular matrix remodeling and inflammatory tissue repair. Galuzo et al. showed that activation of CLEC5A, which is a pattern recognition receptor on myeloid cells, following mRNA COVID-19 vaccination was associated with an enhanced humoral and cellular immune response in a murine model (22). CLEC5A upregulation can contribute to a “trained” immunity-like state in monocytes, as shown in a study by Qi et al., where epigenetic reprogramming of CLEC5A in vascular disease led to persistent monocyte/macrophage dysfunction and heightened inflammatory responsiveness (23). In theory, trained immunity could represent a potential mechanistic pathway underlying the combined effects of HCA and oxygen exposure in monocytes. However, to substantiate this hypothesis, it would be imperative to establish the occurrence of epigenetic reprogramming, functional cellular changes, and the persistence of these effects over time (24). FCGR3A (CD16A) and FCGR2B (CD32B) are monocyte receptors with activating and inhibiting effects on monocytes, respectively (25). In autoimmune disease models, CD101 was shown to be expressed during monocyte differentiation, with an increased LPS-induced pro-inflammatory cytokine production following CD101 engagement (26). PRAM1 is a myeloid-enriched intracellular adaptor protein that is a marker of myeloid differentiation and is associated with an activated innate immune state (27). *FN1* encodes fibronectin, which is a critical component of the extracellular matrix and is involved in cell adhesion, migration, proliferation, and tissue organization. *FN1* upregulation is associated with profibrotic CD14⁺ monocytes and macrophages in systemic sclerosis, where TGF-β signaling drives increased fibronectin production and fibrotic tissue remodeling (28). In contrast, *IL1RN*, *IL7R*, and *HLA-DQB1* were downregulated in HCA–Normoxia. *IL1RN* encodes the interleukin-1 receptor antagonist (IL-1Ra), whereas *IL7R* encodes the interleukin-7 receptor alpha chain, and *HLA-DQB1* encodes the *β*-chain of the HLA-DQ major histocompatibility complex class II molecule. *IL1RN* downregulation could signify less restraint of IL-1-mediated inflammation (29). IL7R can have increased expression in LPS-induced inflammation and plays a role in chronic inflammation and autoimmunity (30). HLA-DQB1 forms a heterodimer expressed on antigen-presenting cells (APCs), such as monocytes, macrophages, B cells, and dendritic cells. HLA-DQB1 has been shown to play a role in immune modulation in patients with severe systemic inflammation, and its downregulation may be associated with impaired antigen presentation and monocyte dysfunction described in severe systemic inflammation and sepsis (31, 32). This may represent another potential pathway underlying the increased risk of sepsis following HCA in the newborn (33); however, further study is needed to establish the significance of this transcription change in monocytes in the context of neonatal sepsis. Taken together, these transcriptional changes suggest a phenotype characterized by monocyte activation and differentiation, reduced anti-inflammatory regulation, impaired antigen presentation, and enhanced extracellular matrix remodeling and pro-fibrotic signaling in the HCA–Normoxia group compared with the No HCA–Normoxia group.

In the No HAC–Hyperoxia group compared with the normoxia group (Table 1B), several immune function-related genes were upregulated, including *CXCL8/IL-8*, *TM4SF19*, and *PLPP3*. CXCL8, also known as interleukin-8, is a pro-inflammatory chemokine produced by monocytes and macrophages in response to inflammatory stimuli. It plays a central role in innate immunity by promoting leukocyte recruitment and activation at sites of inflammation. It has been identified as a biomarker for hyperoxia-induced pulmonary inflammation (34). CXCL8/IL-8 upregulation has been linked to a “trained” immune response—a pathway for innate immune memory involving epigenetic and metabolic reprogramming, followed by enhanced immune response on subsequent injury in lung disease models (35). TM4SF19 (lysosomal membrane protein) and PLPP3 (dephosphorylation enzyme) act as checkpoints or “brakes” for macrophage-driven inflammation (36, 37). TM4SF19 upregulation may also induce a state of sustained dysregulation in lipid-associated macrophages with activation of lysosomal shift and functional reprogramming, thereby contributing to obesity-induced inflammation (38). Together, this shows the possible role of hyperoxia in modulation of the innate immune response in monocytes, which could result in persistent inflammation and chronic disease.

In the No-Hyperoxia group compared with normoxia, key genes involved in monocyte pattern recognition of viruses and bacteria downregulation, including *FPR3* (encodes a G protein-coupled receptor), *TLR 8* (encodes an intracellular receptor), and *TLR 6* (encodes a cell surface receptor). PECAM1 (transmembrane glycoprotein)—which is involved in monocyte survival, maintenance, and functional integrity—was also downregulated. Other studies have also shown the role of FPR3 and PECAM1 in oxidative stress-mediated inflammatory signaling pathways and endothelial cellular injury, respectively (39, 40). In neonatal hyperoxia, downregulation of these genes may bring about dampened immune response to subsequent pathogen exposure and mediate cellular injury. However, further studies are needed to elucidate the biological significance of this transcriptional signature and its role in immune dysfunction.

In the monocytes from the HCA–Hyperoxia group compared to No HCA–Normoxia group, there was a more amplified immune expression, as stated in Table 1C. Genes associated with altered immune function which were upregulated included *CLEC5A*, *FN1*, *DAB2*, and *CCL24*. Activation of *CLEC5A*, *FN1*, and *CCL24* has been linked to a pro-inflammatory state characterized by monocyte/macrophage activation, immune cell recruitment, and amplification of inflammatory signaling (41–43). *DAB2* typically mediates immunosuppression (44). *DAB2* encodes a multifunctional adaptor protein that is involved in clathrin-mediated endocytosis, signal transduction, cell positioning, immune regulation, embryonic endoderm formation, and tumor suppression (45). CCL24 is a protein that is a key mediator of fibrosis, eosinophilic inflammation, and allergic diseases. It is a potential therapeutic target in systemic sclerosis, cardiac fibrosis, and certain cancers (46, 47). CCL24 is also implicated in metabolic syndrome-induced hepatic steatosis (48).

Of the downregulated genes in HCA–Hyperoxia compared with No HCA–Normoxia, the downregulation of *USP18* and *TMEM176B* has been shown to associated with loss of immune “braking” and persistent inflammation and carcinogenesis (49, 50). *TMEM176B* encodes an intracellular cation channel that functions as a dual immunoregulator and is expressed primarily in myeloid cells, including dendritic cells and macrophages, where it modulates inflammasome-driven inflammation in human disease contexts (50). Other studies have reported that genetic or medication-induced inhibition of TMEM176B enhances antitumor immunity and improves cancer recovery (51), supporting its role as an immunomodulator in malignancies. USP18 is the primary negative regulator of type I interferon (IFN-*α*/*β*) signaling. Its downregulation mediates systemic inflammation via prolonged STAT1/STAT2 activation, causing pediatric type I interferonopathies (52). *IFIT1* and *IFIT2*, which were also downregulated in the HCA–Hyperoxia group compared with the No HCA–Normoxia group, are key interferon-stimulated genes with critical roles in antiviral defense, autoimmunity, and cancer biology (53–56). OAS3, the primary activator of the OAS3, which is the primary activator of the OAS/RNase L antiviral pathway, has been shown to be critical for innate immune defense activation against a broad range of viral pathogens and interferon-mediated antiviral immunity (57). Lastly, TNFSF10 (Tumor Necrosis Factor Superfamily Member 10) has been shown to selectively induce apoptosis in cancer and has a role in immune surveillance and regulation (58, 59). Thus, monocytes from the HCA–Hyperoxia group exhibited a profoundly dysregulated immune transcriptional profile compared with the No HCA–Normoxia group, with amplified pro-inflammatory and fibrotic pathways and suppression of anti-infectious and immune regulatory mechanisms.

In our study, HCA–Normoxia (Table 1A) and HCA–Hyperoxia (Table 1C) had important overlapping genes, as shown in Table 7. *CLEC5A*, *FN1*, and *GPRIN3* were upregulated in both HCA–Normoxia and HCA–Hyperoxia, with *CLEC5A* increasing 32.34-fold and 79.2-fold, *FN1* increasing 22.64-fold and 28.26-fold, and *GPRIN3* increasing 5.89-fold and 9.33-fold, respectively. Similarly, the overlapping downregulated genes *HLA-DQB1* and *RGPD1/RGPD2* were decreased in both comparisons, with *HLA-DQB1* showing a 5.43-fold reduction in HCA–Normoxia and a 5.51-fold reduction in HCA–Hyperoxia, while *RGPD1/RGPD2* exhibited a 7.26-fold and 5.24-fold decrease, respectively, relative to the No HCA–Normoxia control group. These expression patterns suggest amplified inflammatory signaling (*CLEC5A*), extracellular matrix remodeling (*FN1*), and altered immune regulation and pathogen recognition (*HLA-DQB1*), primarily driven by HCA in this monocyte model. The roles of *GPRIN3* and *RGPD1/RGPD2* in monocyte biology remain poorly characterized and hence the significance of their expression patterns in this study is unclear.

**Table 7:** Gene transcription comparisons between HCA alone and HCA–hyperoxia.

Gene	HCA alone fold change	HCA–hyperoxia fold change	Direction
*CLEC5A*	32.34^a^	79.20^a^	Both up
*FN1*	22.64^a^	28.26^a^	Both up
*GPRIN3*	5.89^a^	9.33^a^	Both up
*HLA-DQB1*	−5.43^a^	−5.51^a^	Both down
*RGPD1/RGPD2*	−7.26^a^	−5.24^a^	Both down
*CYP1B1*	1.56^b^	4.04^a^	Both up
*DSC2*	1.61^b^	1.65^a^	Both up
*HLA-DQB1*	−7.18^b^	−5.51^a^	Both down
*IVNS1ABP*	−2.55^b^	−2.02^a^	Both down
*PGLYRP1*	−1.51^b^	−1.57^a^	Both down
*VNN2*	1.7^b^	2.09^a^	Both up

a Fold change reported in the present study for the corresponding gene.

b Fold change reported in previous study by Gayen et al. (15)*,* for the corresponding gene.

TNFSF10 (commonly known as TRAIL or TNF-related apoptosis-inducing ligand) emerged as the most important overlapping gene between the No HCA–Hyperoxia (fold change −6.37, Table 1B) and HCA–Hyperoxia groups (fold change −4.26, Table 1C), being consistently downregulated in both comparisons. This finding suggests that hyperoxia exposure, irrespective of HCA status, may suppress TNFSF10-associated signaling pathways in monocytes. In studies on human and animal lung tissue hyperoxia models, lower TRAIL levels were associated with increased inflammation, apoptosis, and lung injury (60), but the significance of its downregulation in monocytes needs further investigation.

We also compared transcriptome signatures in HCA–Hyperoxia exposed monocytes from our study, with the gene expression in monocytes exposed to HCA alone, as reported by Gayen et al. (15) and shown in Table 7. Among the genes with ≥1.5-fold change and *p* ≤ 0.05 in both studies, six genes showed change in the same direction for both HCA alone and HCA–Hyperoxia groups (three upregulated and three downregulated). Of significance in terms of monocyte function, *HLA-DQB1* and *PGLYRP1* were both concordantly downregulated and *VNN2* was concordantly upregulated in both studies. PGLYRP1 (peptidoglycan recognition protein 1) is a pattern recognition protein that plays a role in antibacterial defense and neuroinflammation (61, 62). As we have described earlier, HLA-DQB1 is critical for pathogen recognition and immune activation, and its downregulation in both HCA alone and HCA–Hyperoxia could be a contributory factor to infection risk in exposed neonates; however, this requires further study. On the other hand, VNN2 or Vanin-2, which is a GPI-anchored pantetheinase expressed on monocytes and neutrophils, facilitates monocyte transendothelial migration during inflammation (63, 64). It is concordantly upregulated in both studies—a potential signal of enhanced monocyte recruitment and pro-inflammatory activity.

From Table 2, pathway analysis of HCA–Normoxia versus No HCA–Normoxia (control) revealed enrichment of neutrophil degranulation, mitochondrial dysfunction, oxidative phosphorylation, respiratory electron transport, and class I MHC-mediated antigen processing and presentation pathways, suggesting possible activation of innate immune responses accompanied by alterations in cellular metabolism, mitochondrial function, and antigen presentation. The important canonical pathways implicated in the No HCA–Hyperoxia group include the SUMOylation of DNA damage response. SUMOylation increases after DNA damage and is essential for homologous recombination by promoting DNA end resection and regulating multiple downstream repair proteins, thereby facilitating efficient double-strand break repair. Activation of this pathway is one of several key post-translational modifications in response to stressors such as hypoxia/reoxygenation and can attenuate cellular injury related to both hypoxia and free oxygen radical exposure (65, 66). The sirtuin signaling pathway was also implicated, affecting monocyte and macrophage function. Sirtuins represent potential therapeutic targets for modulating age- and stress-related cellular responses that are associated with cardiovascular disease, dysregulated lipid and glucose metabolism, and altered susceptibility to infections (67). In the HCA–Hyperoxia group, neutrophil degranulation and Class I MHC-mediated antigen processing and presentation pathways were implicated, suggesting the possibility of an alteration in cellular immune response to pathogens and innate immune dysfunction in this model, which could then result in impaired host defense mechanisms.

Results from IPA for upstream regulators in the HCA–Normoxia group compared with No HCA–Normoxia include predicted activation of important pro-inflammatory signaling molecules such as IFNG, TNF (tumor necrosis factor), CSF1 (colony stimulating factor 1) and LPS signaling pathways, suggesting a possible pro-inflammatory myeloid response (Table 4). Upstream regulator analysis in hyperoxia alone revealed activation of TREM-1 and GSK – J4. TREM-1 promotes inflammatory injury by driving mTOR/HIF-1*α* (mechanistic Target of Rapamycin/ Hypoxia-Inducible Factor 1 alpha)-mediated metabolic reprogramming toward glycolysis, which enhances NLRP3 (NOD-like Receptor Pyrin Domain-Containing Protein 3) inflammasome activation and macrophage inflammation; inhibition of this pathway attenuates acute lung injury (68). GSK – J4 is a histone demethylase inhibitor that mediates epigenetic modification through inhibition of JMJD3/UTX (Jumonji Domain-Containing Protein 3/ Ubiquitously Transcribed Tetratricopeptide Repeat, X Chromosome) activity and has demonstrated a therapeutic benefit in malignancies (69).

An important observation in this model was the variable upstream regulator effect of the medication dexamethasone. Dexamethasone is a medication used both prenatally (in the mother) and postnatally (in the baby) to mitigate lung disease and other extra-pulmonary outcomes in preterm infants, but the effect of HCA on its efficacy in the neonate has been shown to be variable and context dependent (70). In our model, hyperoxia alone had a negative influence, but both HCA alone and HCA followed by hyperoxia predicted an activated state for the upstream regulator for dexamethasone, suggesting several biological factors at play in dexamethasone response patterns in the neonate. Consistent with this, clinical studies have indicated that the presence of chorioamnionitis modulates the response to postnatal dexamethasone in preterm neonatal lung disease (71). Several other pro- and anti-inflammatory upstream regulators were also activated in HCA–Hyperoxia, such as lipopolysaccharide, TNF, IFNG, and TGFB1, which may further alter monocyte responsiveness to infection and inflammation in our double-hit disease model.

IPA also revealed that compared with the control group, HCA exposure under normoxia was associated with enrichment of cardiotoxicity, hepatotoxicity, and nephrotoxicity pathways, particularly liver hyperplasia/hyperproliferation, hepatocellular carcinoma, and renal necrosis/cell death. Hyperoxia alone also produced similar toxicological signatures. The combined HCA–Hyperoxia group exhibited enrichment of pathways related to cardiac infarction and enlargement, liver hyperplasia/hyperproliferation, hepatocellular carcinoma, renal necrosis/cell death, and kidney failure (Tables 5A–C). This is reflected in some studies showing the association of perinatal inflammation and/or hyperoxia exposure with cardiac, hepatic, and renal injury in various neonatal models (19, 20, 72). However, additional translational studies are needed to further elucidate the mechanistic pathways linking these exposures to end organ damage and the role of monocytes in disease pathogenesis.

Lastly, IPA identified distinct disease and function networks across the experimental groups (Table 6 and Figures 2A–C). In the HCA–Normoxia group, the highest-ranked networks were associated with developmental disorders, hereditary disorders, neurological disease, molecular transport, and cellular organization. Hyperoxia exposure alone was characterized by networks related to cardiovascular disease, metabolic disease, inflammatory disease, and organismal injury. In the combined HCA–Hyperoxia group, the top networks were enriched for vitamin and mineral metabolism, molecular transport, lipid metabolism, developmental disorders, cardiovascular disease, and organismal injury. Collectively, these findings suggest the possibility that perinatal inflammatory and hyperoxic exposures could activate molecular pathways linked to cardiovascular and metabolic dysfunction, particularly those involving lipid metabolism and systemic injury responses.

This study has several limitations. We did not perform independent qPCR or immunoblot assays to validate transcriptome results. We also did not study postoxygen exposure cellular changes— such as changes in cell morphology, cell viability, cell recovery, apoptosis measurements or RNA yield—after 18–20 h of incubation in hyperoxia or normoxia, which is a significant limitation. In addition, we did not attempt to establish associations between clinical and demographic variables such as sex, mode of delivery, and severity of clinical illness and transcriptome signatures due to the small sample size in this study, therefore limiting power to detect meaningful associations in this regard. We were unable to adjust for donor variability. As a pilot study, the primary purpose was exploratory and focused on feasibility. These results are therefore only hypothesis-generating in nature, and larger, well-powered studies are needed to validate these initial observations. Additional experiments to further understand the role of monocytes in neonatal inflammation should include *ex vivo* tissue coculture or *in vivo* animal validation experiments to study the effect of sequential chorioamnionitis and hyperoxia exposure. Direct comparison between monocytes exposed to HCA alone and HCA–Hyperoxia may provide important information regarding the interaction between the two stimuli. Cellular functional assays such as dexamethasone intervention rescue tests in monocyte cell cultures may further characterize the complex molecular mechanisms of drug response in the setting of inflammation. Lastly, to further our understanding of the implications of this noxious stimulus exposure to long-term monocyte function and immunological memory via the trained immunity pathway, it is essential to investigate for epigenetic reprogramming, demonstrate functional differences by cytokine and other protein assays and demonstrate lasting change in this disease model.

## 5 Conclusions

In summary, this largely unexplored double-hit model in monocytes from term neonates—perinatal inflammation from HCA followed by hyperoxia—revealed a distinct pattern of monocyte gene expression. Hyperoxia alone showed a mixed innate immune activation state with possible impairment of pathogen recognition and immune regulation. In contrast, both HCA alone and HCA–Hyperoxia were associated with amplification of pro-inflammatory and profibrotic signaling, together with dysregulation of antigen presentation and other anti-infectious pathways. In addition, enrichment of cardiometabolic, lipid, endocrine, and organismal injury networks in both exposure conditions was observed. These results provide mechanistic insights into the role of altered monocyte gene expression in neonatal inflammation, which could play a role in mediating diseases like sepsis, chronic lung disease, cardiovascular and metabolic disease in neonatal survivors exposed to HCA and hyperoxia at birth. However, further translational studies are needed to clarify the biological significance of these transcriptional changes and to define their contribution to disease development and long-term outcomes. Due to the small sample size of this pilot study, these findings are only hypothesis-generating and support the need for a larger study to investigate the effects of the double-hit injury of HCA and postnatal hyperoxia on preterm and term infants.

## Data Availability

The original contributions presented in the study are publicly available. This data can be found here: https://www.ncbi.nlm.nih.gov/geo/query/acc.cgi, GEO Accession viewer/accession number GSE346094.
