# Supplementary material for: Sequential exposure to histological chorioamnionitis and postnatal hyperoxia alters gene expression in cord blood monocytes
Source: Front Pediatr. 2026 Sep 15;14:1896464. doi: 10.3389/fped.2026.1896464 (PMC13619921; doi:10.3389/fped.2026.1896464)
Supplement: Supplementary file 1 [file Table1.docx]

**Supplementary Table 1. Genes with altered expression in all three comparison groups**

| **Gene Symbol** | **HCA-Normoxia vs No-HCA-Normoxia** | | **No-HCA-Hyperoxia vs No-HCA-Normoxia** | | **HCA-Hyperoxia vs No-HCA-Normoxia** | |
| --- | --- | --- | --- | --- | --- | --- |
|  | **Fold Change** | **p-value** | **Fold Change** | **p-value** | **Fold Change** | **p-value** |
| **ACTR3C** | -1.67 | 0.009 | -1.51 | 0.026 | -1.87 | 0.003 |
| **ASUN** | 1.9 | 0.042 | 1.77 | 0.027 | 2.05 | 0.015 |
| **ATG16L1** | -1.89 | 0.004 | -1.81 | 0.041 | -1.89 | 0.008 |
| **ATP13A3** | 2.12 | 0.029 | 2.52 | 0.043 | 2.43 | 0.002 |
| **AZGP1** | 1.62 | 0.000 | 1.65 | 0.000 | 1.75 | 0.000 |
| **BTBD10** | -2.01 | 0.005 | -1.73 | 0.049 | -2.33 | 0.002 |
| **CD83** | -2.4 | 0.030 | 1.81 | 0.028 | 1.56 | 0.034 |
| **CDKL5** | -1.62 | 0.028 | -1.57 | 0.029 | -1.72 | 0.005 |
| **COPZ1** | 1.59 | 0.013 | -1.54 | 0.043 | 1.76 | 0.049 |
| **CSF1R** | 2.88 | 0.030 | -3.82 | 0.034 | 2.68 | 0.042 |
| **DROSHA** | -1.96 | 0.002 | -1.57 | 0.020 | -1.74 | 0.006 |
| **EIF4EBP2** | 1.83 | 0.030 | -3 | 0.040 | 1.9 | 0.010 |
| **FAM20A** | -1.69 | 0.004 | -1.99 | 0.001 | -1.85 | 0.002 |
| **FAM151B** | 1.87 | 0.043 | 1.8 | 0.030 | 1.86 | 0.028 |
| **GGH** | 1.99 | 0.000 | 1.76 | 0.007 | 3.61 | 0.000 |
| **HIST1H4L** | -1.71 | 0.026 | -2.38 | 0.015 | -2.28 | 0.002 |
| **HMG20A** | -1.75 | 0.021 | -1.58 | 0.016 | -2.02 | 0.004 |
| **ITGAX** | 1.58 | 0.014 | 1.73 | 0.004 | 2.51 | 0.000 |
| **LRRK2** | 3.84 | 0.001 | -2.53 | 0.030 | 2.51 | 0.010 |
| **MED23** | -1.6 | 0.033 | -1.85 | 0.048 | -2 | 0.004 |
| **MORC2** | 1.62 | 0.012 | 1.59 | 0.035 | 1.82 | 0.002 |
| **NARF** | -1.6 | 0.022 | -1.71 | 0.010 | -1.86 | 0.004 |
| **NSUN4** | 2.07 | 0.003 | 2.03 | 0.012 | 2.78 | 0.000 |
| **OTUD6B** | -2.33 | 0.010 | -1.88 | 0.037 | -3.21 | 0.000 |
| **PLCB1** | 1.9 | 0.014 | 2.05 | 0.006 | 2.31 | 0.003 |
| **PPARG** | 1.7 | 0.047 | 2.03 | 0.015 | 2.2 | 0.002 |
| **RNF8** | -3.15 | 0.001 | -2.17 | 0.035 | -2.73 | 0.000 |
| **SBF2** | 3.23 | 0.000 | -1.9 | 0.020 | 1.98 | 0.029 |
| **SLC25A19** | 3.03 | 0.005 | -2.18 | 0.046 | 2.14 | 0.008 |
| **SUCLG2** | -1.81 | 0.000 | -1.54 | 0.003 | -2.11 | 0.000 |
| **THBS1** | 6.33 | 0.008 | -2.18 | 0.021 | 5.08 | 0.031 |
| **TLR6** | 3.35 | 0.004 | -4.39 | 0.043 | 2.24 | 0.021 |
| **UBE2J2** | 1.88 | 0.010 | -1.82 | 0.032 | 1.95 | 0.006 |
| **UBQLNL** | -1.61 | 0.008 | -1.85 | 0.001 | -1.65 | 0.007 |
| **YIPF3** | 1.67 | 0.023 | -2.23 | 0.037 | 1.98 | 0.016 |
| **ZNF304** | -1.72 | 0.020 | -1.76 | 0.018 | -2.61 | 0.000 |
